# Photoresponsive Electrospun Fiber Meshes with Switchable Wettability for Effective Fog Water Harvesting in Variable Humidity Conditions

**DOI:** 10.1021/acsami.3c07044

**Published:** 2023-08-09

**Authors:** Gregory Parisi, Piotr K. Szewczyk, Shankar Narayan, Urszula Stachewicz

**Affiliations:** † Faculty of Metals Engineering and Industrial Computer Science, AGH University of Krakow, al. A. Mickiewicza 30, Krakow 30-059, Poland; ‡ Department of Mechanical, Aerospace, and Nuclear Engineering, Rensselaer Polytechnic Institute, 110 8th Street, Troy, New York 12180, United States

**Keywords:** switchable, electrospinning, fog capture, PVDF, wetting, water, TiO_2_

## Abstract

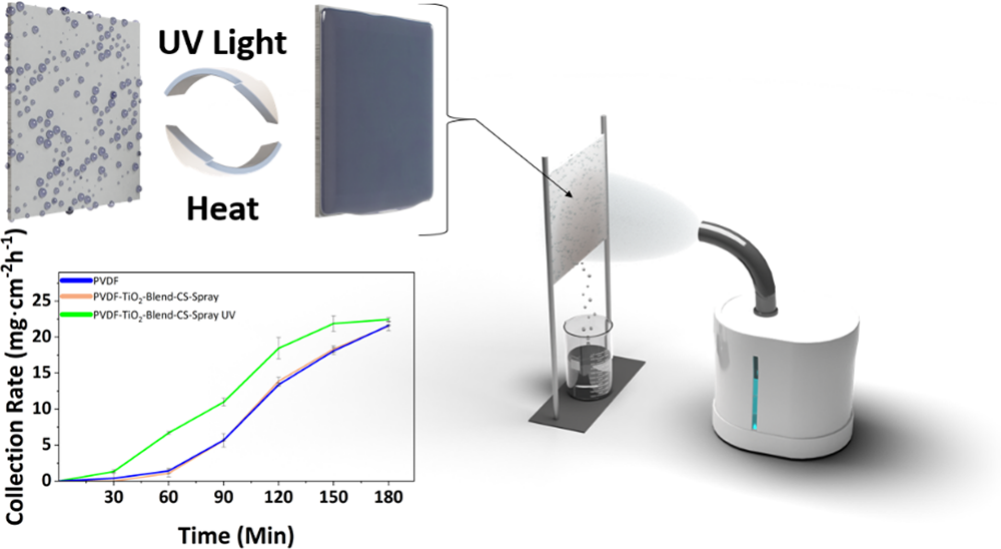

The global water supply worsens yearly with climate change; therefore, the need for sustainable water resources is growing. One of them is fog water collectors with variable surface wettability, with multifunctional designs for utilization worldwide and to address regions with low humidity levels. Therefore, we created fiber meshes with a photoresponsive switchable surface. This study uses electrospun polyvinylidene fluoride (PVDF) meshes, whose wettability is controlled by adding TiO_2_. The fog water collection performance is studied at high and low humidity levels. With TiO_2_-PVDF, the electrospun mesh can be converted from hydrophobic to hydrophilic under UV irradiation and transformed back to a hydrophobic state with heat treatment. The switchable meshes were found to be more effective at water collection after UV irradiation at lower fog rates of 200 mL·h^–1^. The ability to switch between hydrophobic and hydrophilic properties as needed is highly desired in fog collection applications using electrospun meshes, as it can improve overall efficiency after UV irradiation.

## Introduction

Globally, many regions are struggling with water shortages, and innovative technologies are necessary to address the global water supply issue. Water scarcity affects more than two-thirds of the human population and is projected to worsen due to climate change, water pollution, and overconsumption.^1^ Many places worldwide are rain deficient and cannot use methods like water desalination^2^ to provide a sustainable water resource. Fog water collection is an inexpensive and effective method to harvest water in water-scarce regions that are foggy and windy.^3^ Fog consists of water droplets ranging between 5 and 50 μm. Fog harvesting is typically carried out by blowing saturated air with water droplets through a fog water collector.^4^ Fog water collectors are usually constructed using permeable polyethylene (PE) or polypropylene (PP) meshes^5^^,^^6^ and vary significantly in yield, ranging between 3 and 75 L·m^–2^ per day.^7^ An efficient fog collector allows the passage of humid air through the permeable mesh collector, recovers most of the liquid from the fog stream, and quickly releases the water from the mesh for storage.^8^

Inspired by the Namib beetle, there have been many functional materials with hybrid wettability for water harvesting.^9^ Such materials containing hydrophobic and hydrophilic properties integrate and balance two competing processes, droplet capture and shedding, significantly improving water-harvesting efficiency.^10,11^ Catching water droplets is inherently difficult on hydrophobic surfaces in arid regions or low-density fog compared to hydrophilic surfaces. On the contrary, hydrophilic surfaces do not shed droplets as quickly and retain more water on the collected mesh instead of evacuating the collected water to a reservoir.^12^ This behavior has been shown on many substrates, including metals,^13^ ceramics,^14^ polymers,^15^ and hydrogels.^16^ Among many hybrid materials, Knapczyk-Korczak et al.^17^ showed that hybrid composite meshes that contain hydrophobic and hydrophilic fibers improve the draining process to increase the collection efficiency of fog water collectors. High-surface-area fog collectors are commonly produced by electrospinning, where nanofibers are made using a voltage applied between a nozzle and a grounded collector.^18^ The high electric force causes the elongation of the polymer solution into a jet, which is further collected as solid solvent-free fibers.^19^ By using electrospun fibers, it is possible to control the fiber diameter, polymer chemical properties, specific surface, and mechanical properties.^20^ Research groups have tailored the wettability of mats or made a combination of hydrophobic and hydrophilic materials to improve water collection efficiency.^21^

Recently, innovative techniques have been utilized to create smart wettable materials that can reversibly switch wetting behavior.^22^ Switchable wettability has emerged as an interesting research direction because of its significance in applications such as self-cleaning,^23^ microfluidic devices,^24^ water collection,^25^ and oil–water separation.^26−28^ Choi et al.^29^ fabricated a dual-wettability surface using 3D assisted printing. They concluded that hierarchically structured surfaces balanced the main performance combination to optimize the laplace pressure, surface area, and drainage efficiency. Lalia et al.^30^ proposed a lubricant-impregnated electrospun nanomat for fog harvesting, which sheds droplets more efficiently by tailoring the adhesive force between the surface and a water droplet. A thermoresponsive electrospun polymer was used by Thakur et al.^31^ as a way to increase the fog water-harvesting efficiency at different temperatures by varying the contact angle of the membrane. Fog water collection varies depending on optimal surface topography, so researchers have developed special topography modulations or imprinted patterning to increase the fog collection efficiency.^32,33^ Du et al*.*^34^ proposed a PLLA electrospun membrane and conducted their fog water capture experiments with a fog velocity of 60 cm·s^–1^. Their water collection experiments resulted in a total water collection of about 400 mg within a 2 h experiment. Other research groups obtain larger harvesting capacity values, at similar fog flow rates, around 500 mg·cm^–2^ h^–1^, such as Uddin et al.,^35^ but do not have the flexibility and capability of a hydrophobic-superhydrophilic switchable surface.

Previously fabricated fibers used for fog water collectors perform experiments with varying humidity and environmental conditions. Therefore, the performance of water capture depends heavily on environmental conditions like fog flow velocity and ambient humidity. Typically, commercial fog water collectors can capture 3–10 L·m^–2^ of water using Raschel mesh.^36,37^ Ganesh et al*.*^38^ also used low fog velocity for the fog capture experiments, 40 cm·s^–1^, which is still about 2 times the velocity used in our experiment. In their experiment, they reached a total fog capture of 81 mg·cm^–2^ h^–1^ using poly(vinylidene fluoride-*co*-hexafluoropropylene) with fluorinated polyhedral oligomeric silsesquioxane. Although the electrospun mesh is superhydrophobic and sheds water quickly into the reservoir, superhydrophobic coatings typically have a critical issue with surface durability^39^ and are very expensive. Most studies focus on harvesting fog from either a hydrophobic or hydrophilic surface.^40^ Some groups have combined the two characteristics to improve collection efficiency.^41−44^ Using the thermoresponsive polymer poly(*N*-isopropyl acrylamide), Thakur and Ranganath^45^ showed the effect of using a switchable surface for fog water collection. Improving the surface hydrophilicity of electrospun meshes using an alkaline treatment showed improved fog collection with more severe alkaline conditions, demonstrated by Yang et al.^46^

In this study, a novel manufacturing approach of electrospinning and electrospraying was conducted to control the amount of titanium dioxide (TiO_2_) incorporated within polyvinylidene fluoride (PVDF) fibers. TiO_2_ has been widely studied due to its interaction with UV light making it an efficient photocatalyst,^47^ antibacterial,^48^ antifouling,^49^ and photoinduced wettability surface.^50^ TiO_2_ has low reactivity, is chemically inert, and is widely used in outdoor applications such as paints, coatings, and pigments.^51^ Electrospun PVDF was previously used in water collection^52^ and is known for its piezoelectric performance and chemical resistivity.^53^ Notably, the photoinduced wettability of TiO_2_ makes the PVDF fibrous mesh switchable between hydrophobic and superhydrophilic.^54^ The switch to hydrophilic under UV light during light fog conditions maximizes the effect of droplet capture, when hydrophobic surfaces typically underperform. In conditions of heavy fog, when droplet capture is less critical, the electrospun mesh can be heated to revert to a hydrophobic state, thereby maximizing droplet shedding. By investigating the water collection before and after UV irradiation, we found that the two different wetting states affect the water collection efficiency based on the fog rate.

## Experimental Methods

### Materials and Solution Preparation

For the blend and core in coaxial electrospinning solution, a 20 wt % solution, TiO_2_ (Sigma-Aldrich, U.K., particle size <100 nm) was dissolved in dimethylacetamide (DMAc, Sigma-Aldrich, U.K.) and acetone (Sigma-Aldrich, U.K.) in a 1:1 ratio. The solution was sonicated for 1 h in an ultrasonic bath (Bandelin, Sonorex, Germany). Then, 24 wt % of polyvinylidene fluoride (PVDF, Sigma-Aldrich, U.K., *M*_w_ = 350,000 g·mol^–1^) was added to the above mixture. The solution was stirred at 400 rpm for 4.0 h on a hot plate set to 50 °C (IKA RCT basic, Staufen, Germany). For the shell in coaxial electrospinning and electrospraying solutions, 20 wt % of TiO_2_ was dissolved in DMAc and acetone in a 3:1 ratio. The solution was then further sonicated in an ultrasonic bath for 2 h prior to use.

### Electrospinning

**Figure 1:**
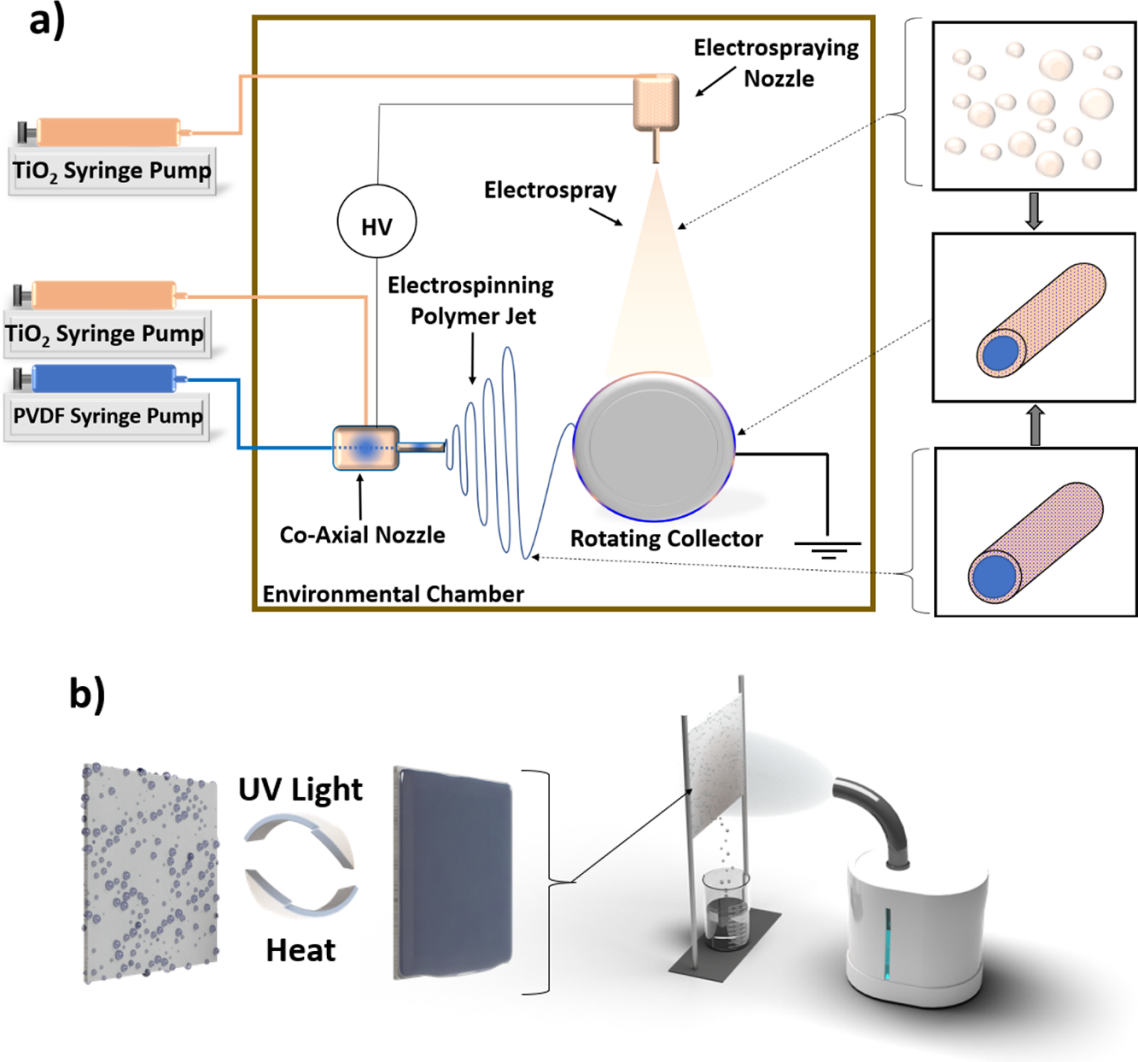
(a) Schematic of electrospinning and electrospraying setup with coaxial nozzle. (b) Fog harvesting schematic with the wettability switch with the addition of UV light or heat.

The fibers were electrospun using an IME Technologies (Waalre, Netherlands) electrospinner with a climate control system. The PVDF-TiO_2_ fiber meshes were produced by applying a voltage of +18 kV to the 19 G stainless needle, which was set to 18 cm distance from the collector. The chamber’s environmental conditions were kept at *T* = 24 °C and a relative humidity (RH) of 60%. The flow rate for the core solution was set to 1.0 mL·h^–1^, the flow rate for the shell was kept constant at 0.1 mL·h^–1^, and the flow rate for the electrospray was 0.5 mL·h^–1^. The electrospraying was done at 14 cm distance between the needle and the collector. The collector was held at a constant rotating speed of 30 rpm. The same conditions were used during electrospinning and electrospraying since they were performed simultaneously. Figure 1a illustrates the environmental chamber with the electrospinning setup and the manufacturing steps of electrospun meshes.

### Surface Characterization

Electrospun samples were analyzed using a scanning electron microscope (SEM, Merlin Gemini II, ZEISS, Germany). Before imaging, the samples were coated with either an 8 nm Au layer for SEM imaging using a rotary pump sputter coater (Q150RS, Quorum Technologies, U.K.) or a 10 nm C layer for EDX using a carbon coater (Emitech K950, U.K). Fiber diameters were measured from SEM micrographs using ImageJ software (1.53 k, NIH, USA). The average fiber diameter values were calculated from 100 measurements, and the error was based on the standard deviation with (2022, OriginLab, USA) software. The chemical composition of the fibers was analyzed on aluminum substrates using energy-dispersive X-ray spectroscopy (EDX, Bruker Quantax 800) and Fourier transform infrared spectroscopy (FTIR, Nicolet, iS-5, USA). The wettability of the fibers was measured by imaging 3 μL volume droplets of deionized water (DI, Spring 5UV purification system, Hydrolab, Poland) repeated seven times using a camera with a macro lens (EOS 700D, EF-s 60 mm, Canon, Japan). Contact angles were measured on a horizontally placed mesh and analyzed using a contact angle plug-in on ImageJ software (1.53, NIH, USA). UV irradiation was performed in air by six parallel UV lamps (9 W, 285 nm) at 4 cm from the electrospun mats. The mat was heated in an oven at 60 °C (Pol-eko Aparatura, Poland) for 2 h. The PVDF films for flat surface contact angle measurements were prepared using spin coating (L2001A v.3, Ossila, Sheffield, U.K.).

### Water-Harvesting Experimental Setup

Water harvesting was conducted on electrospun mats (10 × 10 cm) placed vertically in front of a fog simulator (Beurer GmbH, Germany), as illustrated in Figure 1b. The fog was produced by a humidifier at a flow rate set to 400 mL·h^–1^ with a fog flow velocity of 19 cm·s^–1^ and a constant humidity above 95%. The humidifier was placed 6 cm from the vertical electrospun mat. The water was collected in a beaker placed under the mesh and weighed every 30 min for 3 h, according to previous experiments.^55^ The electrospun mesh was also weighed before and after the water-harvesting experiments. The water collection rate was calculated using eq 1, where *m* is the mass, *A* is the fog collection area on the mat, and *t* is the collection time. To switch the wettability of the fiber mesh from hydrophobic to hydrophilic, the mesh was placed in the UV chamber for 2 h. To switch the wettability of the fiber mesh from hydrophilic to hydrophobic, the mesh was placed the oven for 2 h.
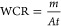
1

## Results and Discussion

### Fiber Morphology and Composition

**Figure 2:**
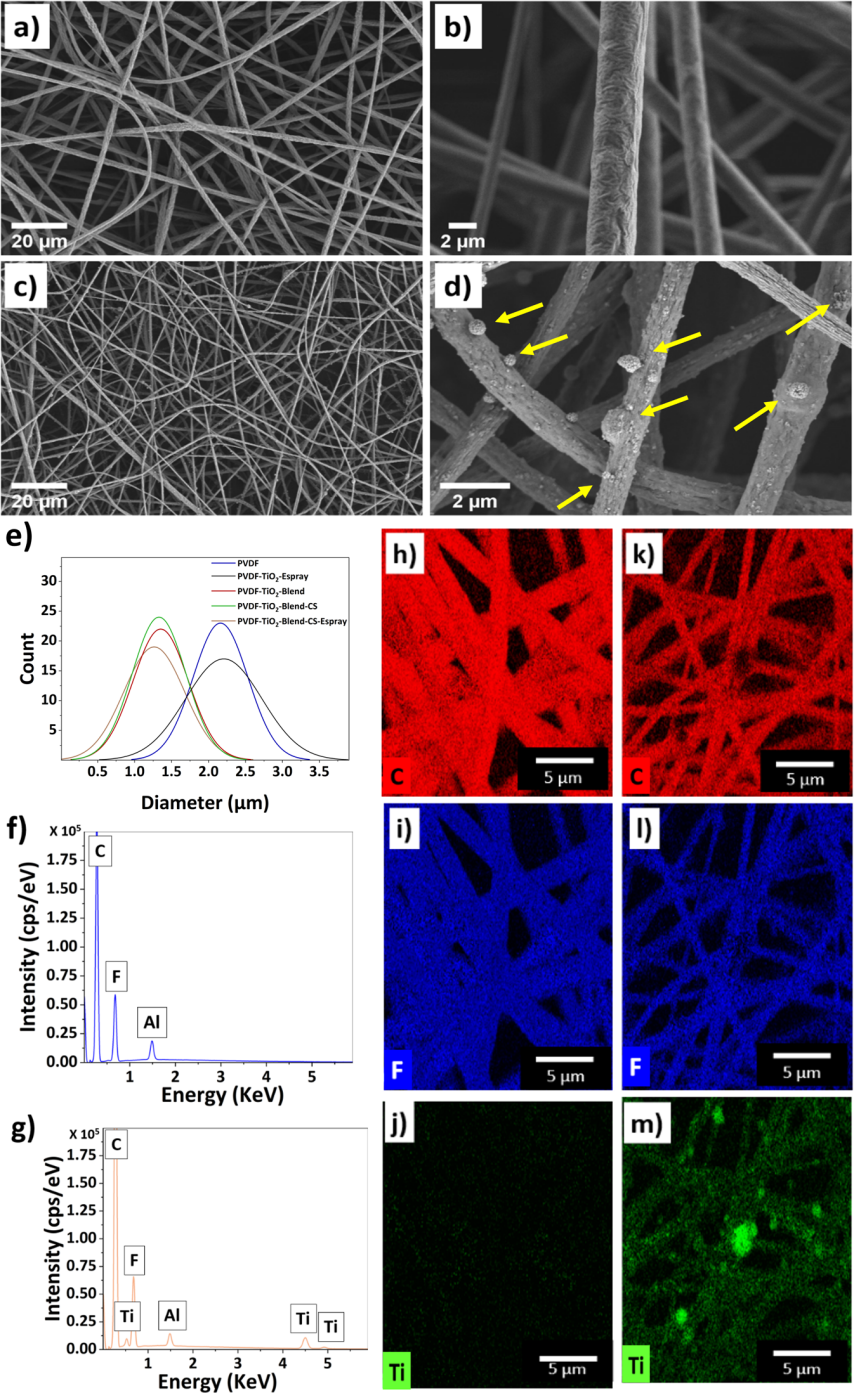
SEM micrographs of (a, b) pristine PVDF fibers and (c, d) PVDF-TiO_2_-Blend-CS-Espray fibers, arrows indicating TiO_2_ nanoparticles, and (e) fiber diameter distributions. EDX spectra from (f) EDX pristine PVDF fibers and (g) PVDF-TiO_2_-Blend-CS-Espray fibers. (h–j) EDX elemental mapping of PVDF fibers and (k–m) EDX elemental mapping of PVDF-TiO_2_-Blend-CS-Espray fibers, carbon indicated in red, fluorine indicated in blue, and titanium indicated in green.

Surface topography and chemical composition play an essential role in fog water collection. The SEM images of PVDF and PVDF-TiO_2_-Blend-CS (Core–Shell)-Espray are shown in Figure 2a–d. The pristine PVDF fibers are generally smooth on the microscale and free of beads or fiber entanglements. The morphology of the fibers possesses a wrinkled surface driven by the high humidity in the electrospinning chamber.^56^ The addition of TiO_2_ changes the morphology of the fibers, which contain large protruding bumps from the blended PVDF-TiO_2_ and small TiO_2_ particles on the outer surface of the fibers present because of the core–shell electrospinning process. Additionally, spherical agglomerations of TiO_2_ scattered throughout the electrospun mesh from the electrospraying technique can be observed (see Figure 2d).

Histograms indicating fiber size distribution are shown in Figure 2e. The average fiber diameters of the pristine PVDF fibers and PVDF-TiO_2_ Espray are 2.2 ± 0.52 and 2.2 ± 0.37 μm, respectively. The average fiber diameter of the previously mentioned two samples does not significantly change because the polymer solution used was the same. The only difference was the addition of TiO_2_ electrospraying, which does not interact with the electrospinning process. This fiber diameter is slightly higher in comparison to prior work on electrospun PVDF, which was, on average, a diameter of 1.29 μm with wrinkled surface topography.^52^ The average fiber diameter for the PVDF-TiO_2_-Blend-CS-Espray fibers is 1.26 ± 0.41 μm. Additional SEM images for the remaining three meshes that were not used for water harvesting can be found in Figure S1 in the Supporting Information. The diameter of PVDF-TiO_2_-Blend-CS-Espray fibers is reduced due to the addition of TiO_2,_ which lowers the entanglement and the spinnability of PVDF chains, decreasing the fiber diameter.^57^ Additional TiO_2_ in the shell of the electrospinning process and TiO_2_ electrospraying does not significantly change the average fiber diameter of the PVDF-TiO_2_-Blend and PVDF-TiO_2_-Blend-CS samples.

EDX analysis was used to verify the elemental composition of the PVDF fibers and the PVDF-TiO_2_-Blend-CS-Espray fibers. Figure 2f shows that the pristine PVDF fibers contain only the elements that are present in the polymer solution of PVDF: C and F. The PVDF-TiO_2_-Blend-CS-Espray sample shows the additional Ti peak confirming the addition of TiO_2_, see Figure 2g. Furthermore, the elemental mapping results in Figure 2h–m indicate the high concentration of TiO_2_ present within the PVDF fiber mat. Elemental Ti was uniformly distributed throughout the PVDF fibers. The pristine PVDF fibers show similar results for the elements C and F; however, Ti is not present. Additional elemental mapping from EDX and FTIR spectrum can be found in Figures S2 and S3 in the Supporting Information.

### Wettability

The wettability of an electrospun membrane is a crucial factor in fog water-harvesting efficiency. The water contact angle is a measure of surface-energy interactions between the droplet and the surface.^58^ PVDF is inherently hydrophobic, and adding roughness due to the addition of TiO_2_ increases the surface area and creates voids,^59^ which further increases the contact angle of a hydrophobic PVDF.^60^ Although surface roughness increases the hydrophobicity of a material, it can also incorporate imperfections and physical defects,^61^ which the Young’s equation does not predict well since it assumes an equilibrium or static contact angle. Contact angle hysteresis (Δθ) is related to the energy required for a droplet to move from one state to another on the same surface. The static contact angle lies between the advancing (θ_A_) and receding (θ_R_) contact angles. The Δθ determines how well a droplet will move, with a smaller hysteresis indicating easier droplet movement. Contact angle hysteresis is shown in Figure S5 and Table S1 in the Supporting Information. With a rough surface, the apparent contact angle is observed. It can take the form of either the Wenzel state or the Cassie–Baxter state (eq 2). Where Θ_r_ is the contact angle on a rough surface, Θ is the contact angle on an ideally smooth surface, *f*_1_ is the fraction of liquid droplet in contact with the solid, and *f*_2_ is the fraction of the droplet in contact with air (*f*_1_ + *f*_2_ = 1).
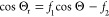
2

**Figure 3:**
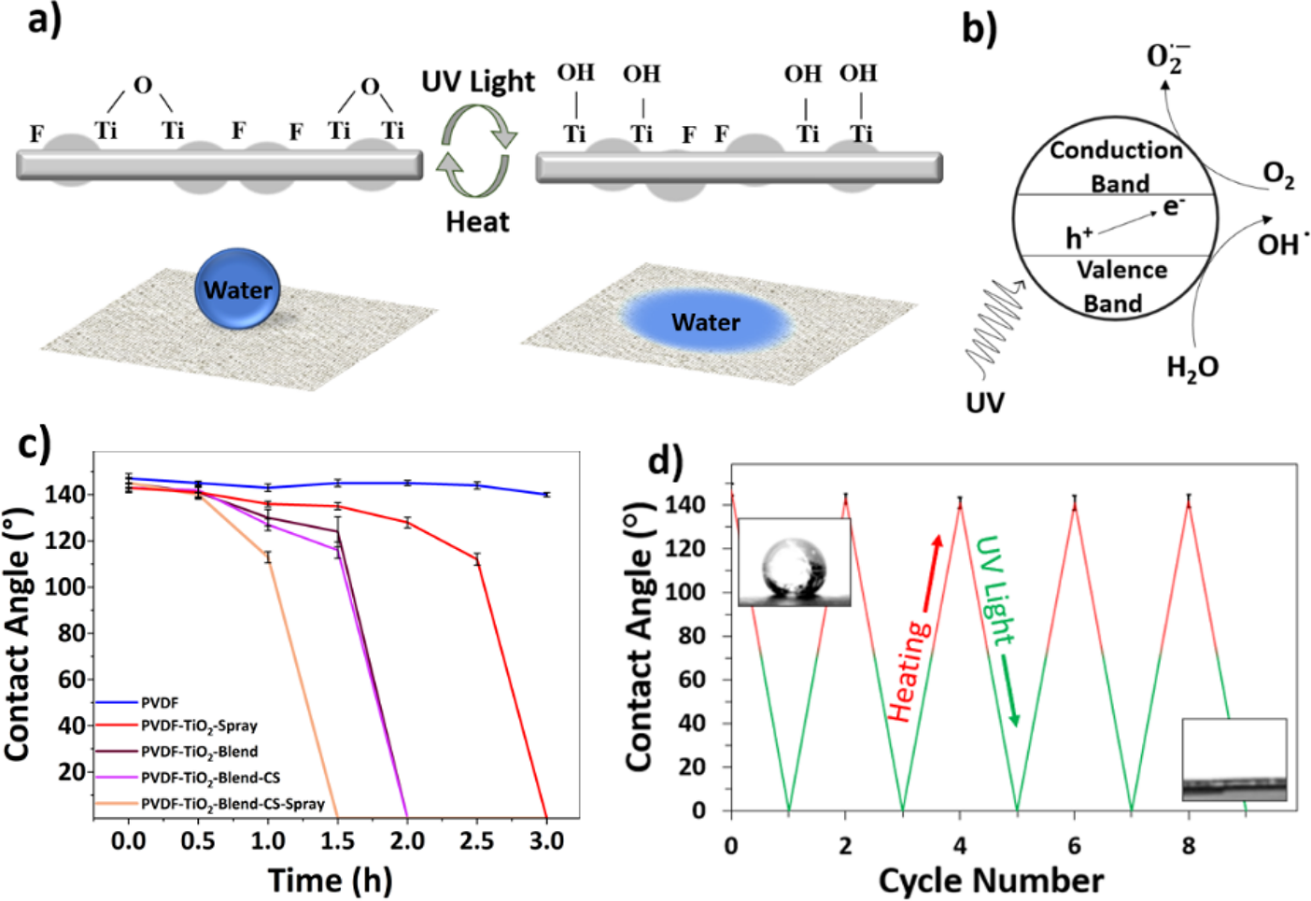
(a) The mechanism of the conversion process of wettability of PVDF+TiO_2_ fibers, (b) the photocatalytic mechanism of TiO_2_ nanoparticles, (c) water contact angles of samples with the varying additive process of TiO_2_ under UV irradiation, and (d) cycling between hydrophobic and hydrophilic with the addition of UV irradiation followed by heat, inserts are water droplet on PVDF-TiO_2_-Blend-CS-Espray mesh before and after UV light.

The static contact angle of pristine PVDF film was measured to be 118 ± 3.7°, and pristine PVDF electrospun fibers were estimated to be 147 ± 2.5°. Accordingly, eq 2 gives *f*_1_ = 0.304 and *f*_2_ = 0.696. The calculation shows that the area fraction of water droplets contacting air within the voids of the fibers is 69.6%. Introducing polar TiO_2_ does not significantly reduce the hydrophobicity of the electrospun PVDF, 145 ± 2.8°. TiO_2_ possesses a photoinduced wetting property, which changes the water contact angle of the surface with exposure to UV light. Photoinduced hydrophilicity causes the surface of a hydrophobic material to become entirely wetted by water. UV irradiation creates surface O vacancies by directly driving surface O from the surface (Figure 3a). The O vacancies separate absorbed water and create a surface populated by hydroxyl groups and turn the surface hydrophilic with additional water. UV irradiation produces photogenerated holes (h+) and electrons (e−) (Figure 3b). The photogenerated holes directly attack the surface Ti–O bonds, breaking them apart in coordination with H_2_O.^62^

The switching UV wettability was demonstrated by measuring the water contact angle along with increasing UV irradiation time (Figure 3c). The sample with the most TiO_2_, PVDF-TiO_2_-Blend-CS-Espray, exhibited a decrease in contact angle and turned superhydrophilic after 90 min of UV irradiation. By contrast, the pristine PVDF fibers showed no response in water contact angle to UV irradiation. The hydrophobicity of the membrane can be recovered with a heating treatment at 60 °C for 2 h. The transition back to hydrophobic surface properties is due to the dehydration process generated by heat and changes the Ti–O–H bonds back to Ti–O bonds. This process was completely reversible and was demonstrated by switching back and forth several times in Figure 3d. The UV switched meshes maintained hydrophilicity for several days (Figure S7 in the Supporting Information), similar to other photocatalysis work using TiO_2._^63^ Before UV irradiation, both PVDF fibers and PVDF-TiO_2_-Blend-CS-Espray fibers show a nearly spherical water droplet shape, which can be found in Figure S4 in the Supporting Information. After UV irradiation, PVDF fibers maintain a spherical shape of water droplets; however, PVDF-TiO_2_-Blend-CS-Espray fibers become completely wetted, with a contact angle close to 0°. It should be noted that not all five manufactured meshes were used for fog water harvesting. Since PVDF-TiO_2_-Blend-CS-Espray fibers showed the quickest response, this was the only TiO_2_ impregnated mesh that was used in fog water collection compared to pristine PVDF fibers.

### Fog Water Collection

**Figure 4:**
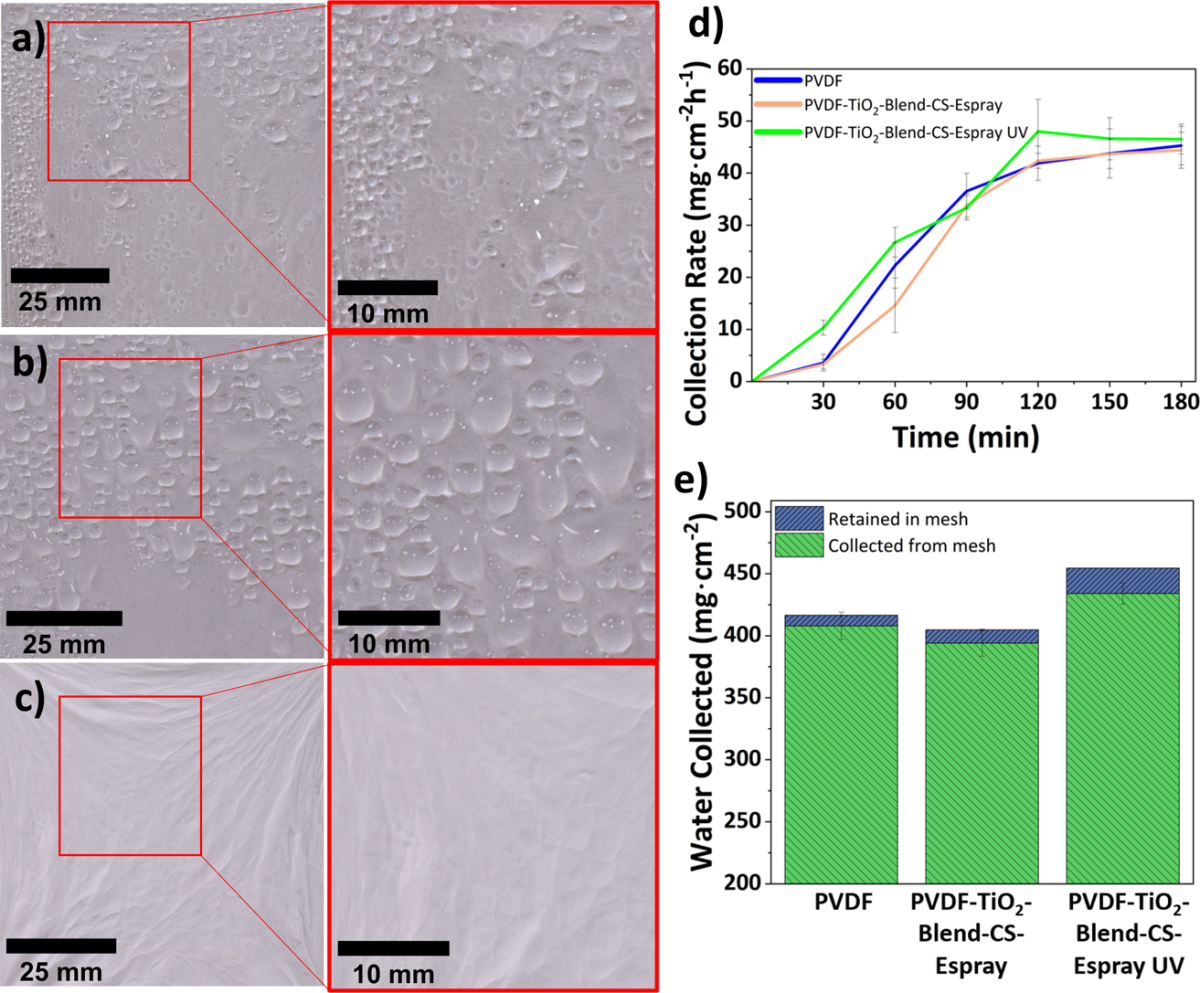
Images of water droplets during fog collection experiment (400 mL·h^–1^) deposited after 60 min on (a) PVDF fibers, (b) PVDF-TiO_2_-Blend-CS-Espray fibers before UV irradiation, and (c) PVDF-TiO_2_-Blend-CS-Espray fibers after UV irradiation, (d) results of water collection rate of all three electrospun mats, and (e) comparison of total water collected from mesh and retained in the mesh after 3 h.

The fog collection behavior on the electrospun mats is shown in Figure 4a–c. Discrete droplets are collected on the PVDF and PVDF-TiO_2_-Blend-CS-Espray mats, which can be seen from the first instances of fog capture to droplet growth and coalescence to the moment the droplet grows large enough to fall off the mesh from the force of gravity.^64^ Since both mats consist of the same polymer materials and similar fiber size, there is not too much difference in discrete droplet capture and growth, as shown in Figure 4a,b. Likewise, since the contact angle hysteresis is similar, see in the Supporting Information, Figure S5 and Table S1, the droplet shedding and collection do not greatly differ between PVDF and PVDF-TiO_2_-Blend-CS-Espray fibrous mats. However, after UV irradiation, the PVDF-TiO_2_-Blend-CS-Espray mats completely change their wetting characteristics and become superhydrophilic. There is a distinct difference in the behavior of droplet capture and growth of the mesh after UV irradiation. As Figure 4c shows that a thin layer of water is captured on the surface of the mesh instead of discrete droplets. This characteristic was observed throughout the entire 3 h fog water-harvesting experiment.

The chemical composition of the fibers, surface geometry, roughness, and wettability of electrospun mats are crucial for fog water harvesting, which is evident within the first hour of water collection. There are discrete droplets throughout the membrane when UV irradiation is not applied. After 3 h on such surfaces, enough droplets roll off and collect on the mesh to create a thick film of water, completely transforming the hydrophobic material to a Wenzel state, where water replaces air within the fibers’ voids. In this case, the droplet-to-air area fraction changes from 69.6 to nearly 0%, which is implied from (eq 2) based on the change in water contact angle from 147 to near 0°.

The transition between Cassie–Baxter and Wenzel state with a switchable surface has been demonstrated for various surface geometries and materials.^65^ In this study, after UV irradiation on the fibers, water droplets quickly spread on the mat’s surface. This enhances the fog water collection process on the mesh after UV irradiation. During fog water harvesting, the photoresponsive membrane can switch to hydrophilic under UV light during light fog conditions to maximize the effect of droplet capture and droplet nucleation, when hydrophobic surfaces typically underperform.^66^ During heavy fog conditions, when droplet capture is not as crucial, the electrospun mesh can be switched back to hydrophobic under heat to maximize droplet shedding.

Figure 4d shows the average water collection trends over the 3 h experiment. After 180 min of fog water collection, the PVDF, PVDF-TiO_2_-Blend-CS-Espray, and PVDF-TiO_2_-Blend-CS-Espray UV-irradiated mats exhibit similar water collection rates, 45.3, 44.4, and 46.5 mg·cm^–2^ h^–1^, respectively. However, the UV-irradiated mesh has a significantly higher collection rate within the first 70 min of harvesting. In the first 30 min, the collection rate is about 3 times higher than both hydrophobic nonirradiated mats. Consequently, the largest amount of water was obtained for PVDF-TiO_2_-Blend-CS-Espray UV, corresponding to a total of 434.0 mg·cm^–2^.

Droplet shedding is also sensitive to the wetting properties of the mats. Typically, hydrophobic mats shed droplets quicker and more efficiently than their hydrophilic counterparts. This can be seen in the amount of water collected from the mesh and retained in the mesh (Figure 4e). Since droplets are not immediately collected in the beaker, all meshes retain water on the mesh after the experiment has concluded. The amount of water retained is 8.6 mg·cm^–2^ for the PVDF mesh, 10.7 mg·cm^–2^ for the PVDF-TiO_2_-Blend-CS-Espray mesh, and 20.5 mg·cm^–2^ for the PVDF-TiO_2_-Blend-CS-Espray UV-irradiated mesh. Water capture and droplet nucleation are more efficient on hydrophilic materials and show a greater water collection rate in the beginning of an experiment, especially in the first 1 h of the experiment. There is a 16% increase in total water capture from the UV-switched PVDF-TiO_2_-Blend-CS-Espray mesh compared to the other two mats. Because of the fast droplet capture kinetics of the UV-irradiated mesh, the total water collected is 6% greater for the PVDF-TiO_2_-Blend-CS-Espray sample.

**Figure 5:**
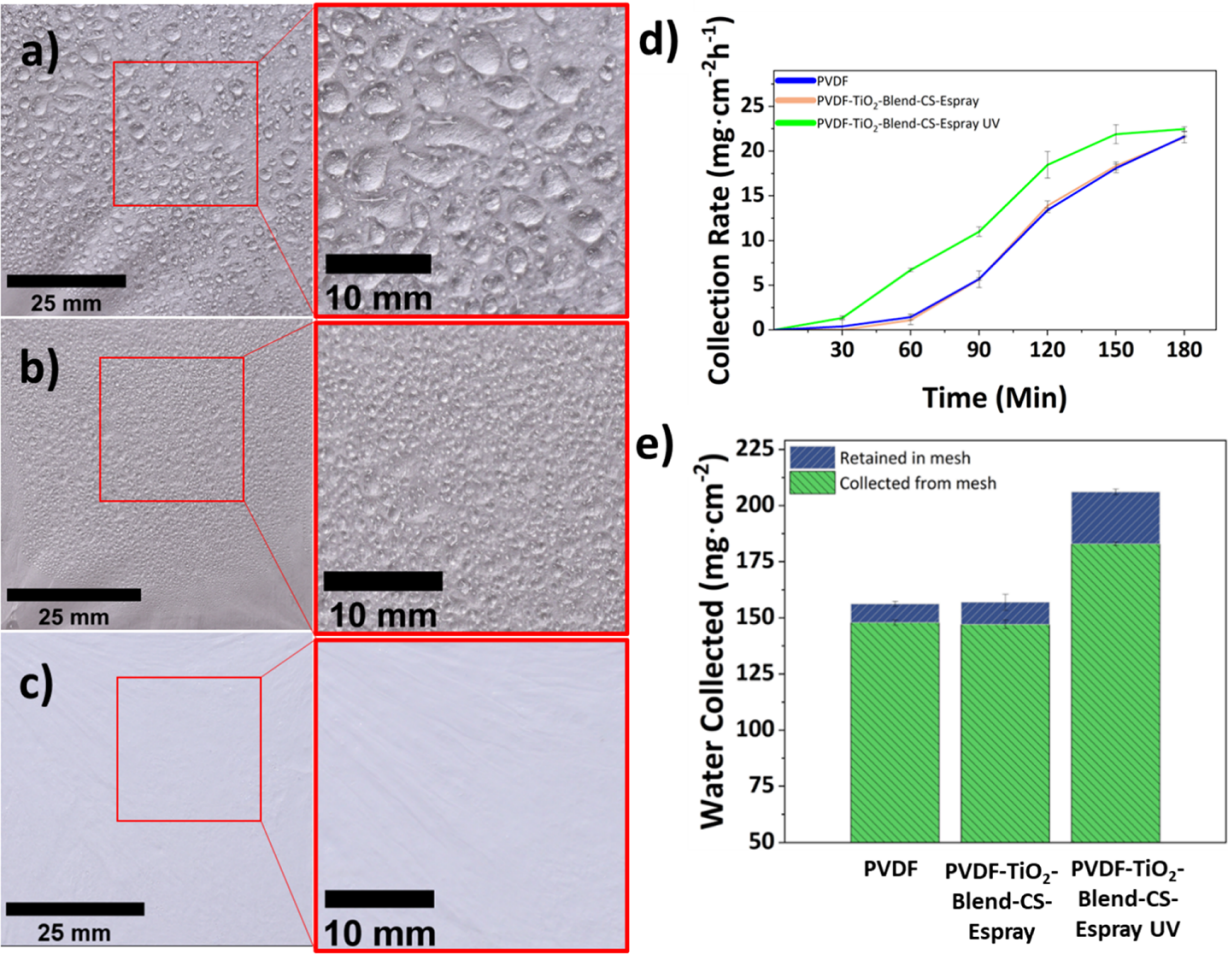
Fog collection on low fog rates after 60 min on (a) PVDF fibers, (b) PVDF-TiO_2_-Blend-CS-Espray fibers before UV irradiation, and (c) PVDF-TiO_2_-Blend-CS-Espray fibers after UV irradiation, (d) water collection rate of all electrospun mats, and (e) total water collected from mesh and retained in the mesh after 3 h.

The difference in the collection rate between the irradiated and nonirradiated samples is more significant in low-fog environments. The same experiment was conducted under lower fog conditions, with a 200 mL·h^–1^ fog flow rate (Figure 5a–e). Similar trends can be seen compared to the higher flow rate with all three samples (Figure 5d). However, after UV irradiation, the PVDF-TiO_2_-Blend-CS-Espray fibers exhibit a more significant collection rate throughout the 3 h experiment. At 30 min, there is no water collected from the nonirradiated samples. At 60 min, the PVDF-TiO_2_-Blend-CS-Espray UV mesh has more than 3 times the water collection rate compared to PVDF-TiO_2_-Blend-CS-Espray and PVDF mats, 6.7, 1.1, and 2.0 mg·cm^–2^ h^–1^. After 90 min of water collection, the irradiated PVDF-TiO_2_-Blend-CS-Espray UV mesh outperforms the other mats by more than double the collection rate, 11.0 mg·cm^–2^ h^–1^. Hydrophobic samples begin to shed droplets more efficiently after enough time, and the collection rate significantly improves. This is because after the droplet is removed from the surface, there are additional sites on the mesh to capture additional water droplets. Interestingly, the amount of water retained is not significantly affected by the change in the humidity flow rate. This is because only a certain amount of water can be retained on the mesh before the water is evacuated into the collection beaker. The amounts of water collected after 3 h for PVDF, PVDF-TiO_2_-Blend-CS-Espray, and PVDF-TiO_2_-Blend-CS-Espray UV mats were 148, 147, and 183 mg·cm^–2^ (Figure 5e). Additionally, the durability of the meshes was investigated in order to find the possibility of functioning in practical applications. Within 9 h of fog collection with a single mesh, the water retention of the mesh did not decrease, demonstrating reusability and durability of the electrospun meshes, see Figure S6 in the Supporting Information.

The current study uses a photoresponsive switchable surface that can mimic a hydrophobic or hydrophilic surface without changing the fog harvesting mat. The addition of TiO_2_ also adds benefits to fog water collection, and the amount of TiO_2_ is entirely controllable, demonstrated by the electrospinning process. Adding TiO_2_ can improve the degradation rate of organic pollutants and contains antibacterial and antifouling properties.^67^ The switching of the surface becomes more prevalent with a large difference in humidity levels, as shown in our results. We show the possibility of adjusting the wettability of meshes to fog conditions to increase the efficiency of water collection mechanisms. Switching materials offer the advantage of using a single material that can switch wetting properties, providing a more cost-effective and versatile solution for fog water collection. The ability to switch before the process begins, based on anticipated fog conditions, allows for optimal water capture.

## Conclusions

In the current work, we investigated the fog water collection on pristine PVDF meshes and PVDF-TiO_2_-Blend-CS-Espray meshes with photoresponsive switchable surface properties. The PVDF-TiO_2_-Blend-CS-Espray meshes were designed and optimized to increase the TiO_2_ concentration in the fibers to switch the surface between hydrophobic and hydrophilic with UV irradiation and heat, respectively. At high humidity levels, the water collection rates after 180 min were similar for PVDF, PVDF-TiO_2_-Blend-CS-Espray, and PVDF-TiO_2_-Blend-CS-Espray UV, 45.3, 44.4, and 46.5 mg·cm^–2^, respectively. However, because of the hydrophilic nature of the PVDF-TiO_2_-Blend-CS-Espray UV meshes, the water collection rate was more than 3 times larger within the first 30 min. The importance of the switchable materials becomes more prevalent at low fog rates of 200 mL·h^–1^. The hydrophilic mesh outperforms the nonirradiated samples throughout the entire 180 min. Twenty-four percent more water was collected from the UV-irradiated mesh, 183 mg·cm^–2^, compared to the next most efficient pristine PVDF fibers, 148 mg·cm^–2^. Fog water collectors with switchable surfaces are important in utilizing the same materials in different atmospheric conditions. Since the surface properties can be tailored, the electrospun mesh does not need to be changed during different parts of the day or different fog conditions since these mats can adopt hydrophobic or hydrophilic properties on demand. In fog collection applications, the efficiency of the electrospun mesh can be maximized during humid or arid conditions.

## Data Availability

The data that support the findings of this study are available from the corresponding author upon reasonable request.

## Supporting Information Available

Figure S1: SEM images of electrospun fibers; Figure S2: EDS Elemental mapping; Figure S3: FTIR spectroscopy; Figure S4: water droplets on electrospun meshes; Figure S5: fog harvesting advancing and receding angles; Figure S6: changes of water retention capacity of meshes; Figure S7: hydrophilicity stability after UV switching; Table S1: advancing and receding water contact angles (PDF)
