# Supplementary material for: Photoresponsive Electrospun Fiber Meshes with Switchable Wettability for Effective Fog Water Harvesting in Variable Humidity Conditions
Source: ACS Appl Mater Interfaces. 2023 Aug 9;15(33):40001–10. doi: 10.1021/acsami.3c07044 (PMC10450686; doi:10.1021/acsami.3c07044)
Supplement: Supplementary file 1 — am3c07044_si_001.pdf [file am3c07044_si_001.pdf]

Supporting Information

Photoresponsive Electrospun Fiber Meshes with Switchable Wettability for Effective Fog Water Harvesting in Variable Humidity Conditions

Gregory Parisi [b], Piotr K. Szewczyk [a], Shankar Narayan [b], Urszula Stachewicz [a]*

[a] AGH University of Science and Technology, Faculty of Metals Engineering and Industrial Computer Science, al. A. Mickiewicza 30, Krakow 30-059, Poland
[b] Rensselaer Polytechnic Institute, Department of Mechanical, Aerospace, and Nuclear Engineering, 110 8th Street, Troy, NY 12180, United States

* Corresponding Author: ustachew@agh.edu.pl

The Supporting Information includes:

- Figure S1: SEM images of electrospun fibers
- Figure S2: EDS Elemental mapping
- Figure S3: FTIR Spectroscopy
- Figure S4: Water droplets on electrospun meshes
- Figure S5: Fog harvesting advancing and receding angles
- Figure S6: Changes of water retention capacity of meshes
- Figure S7: Hydrophilicity stability after UV switching
- Table S1: Advancing and receding water contact angles

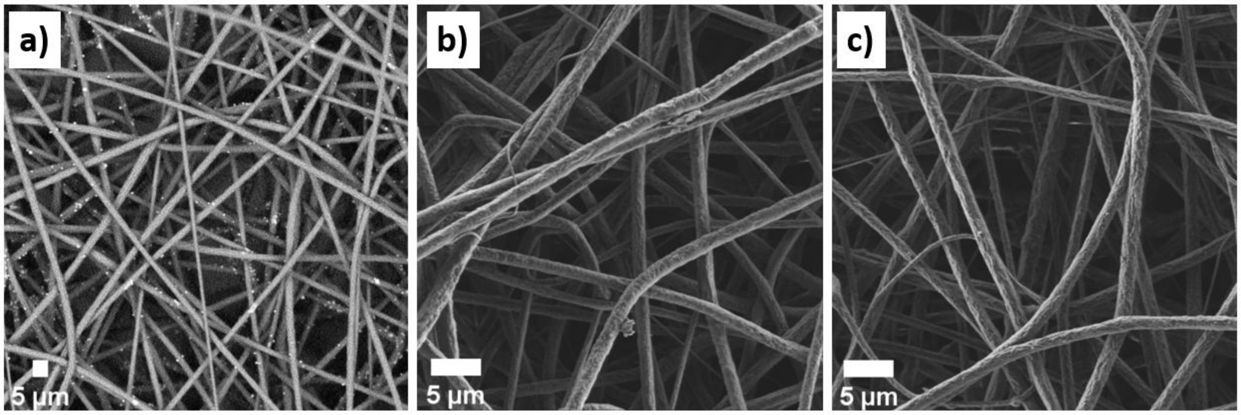

Figure S1. SEM micrographs of (a) PVDF-TiO2-Espray (b) PVDF-TiO_2-Blend (c) PVDF-TiO_2-Blend-CS

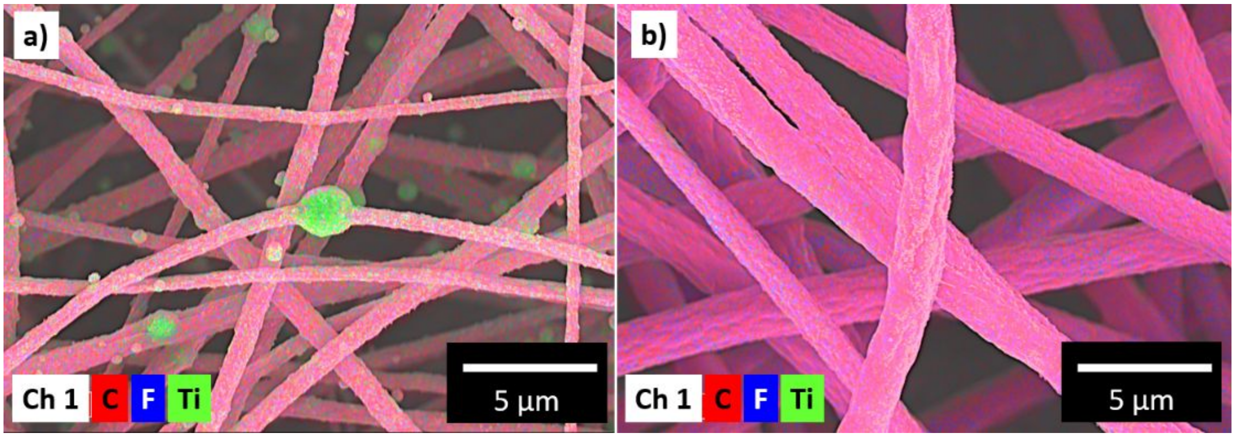

Figure S2. EDS elemental mapping of (a) PVDF-TiO_2-Blend-CS-Espray and (b) PVDF

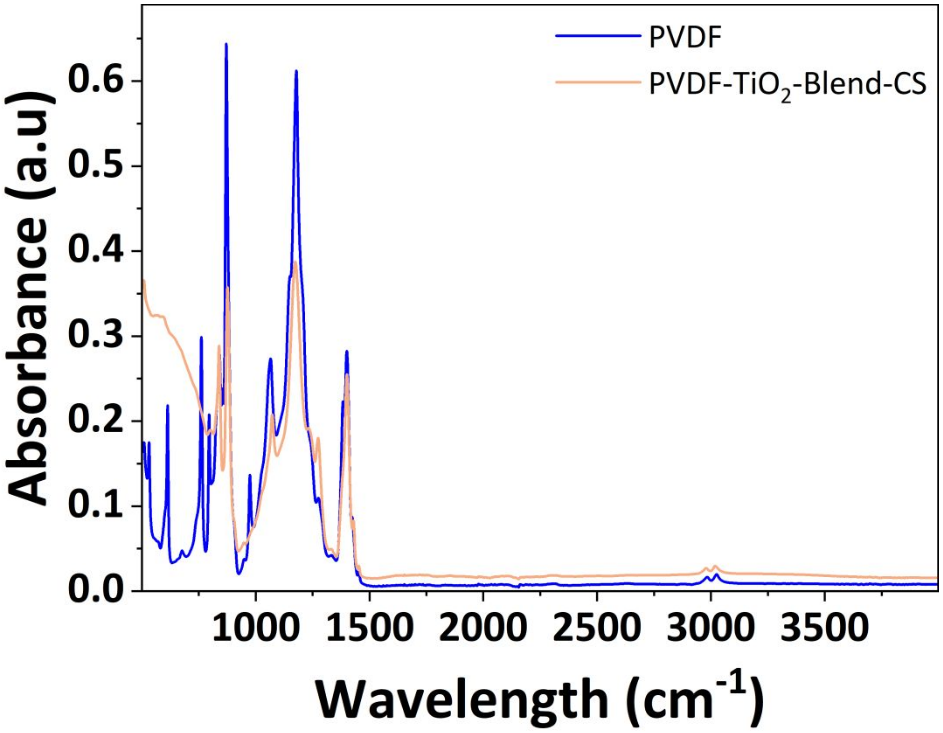

Figure S3. FTIR of PVDF membrane and PVDF-TiO_2-Blend-CS membrane.

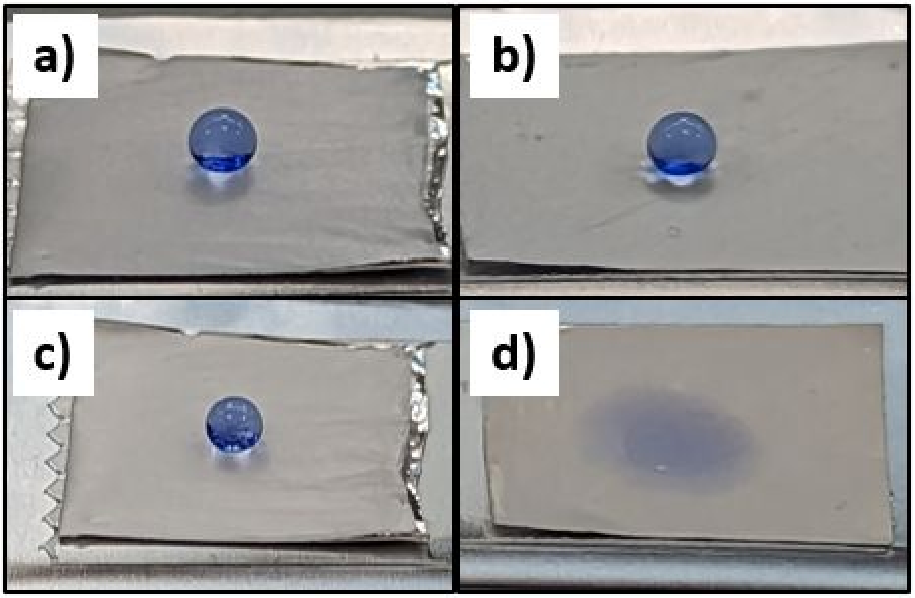

Figure S4. Water contact angle (dyed blue with methylene blue) of (a) PVDF, (b) PVDF-TiO2-CS-Espray, (c) PVDF after UV irradiation, (d) PVDF-TiO_2-CS-Espray after UV irradiation.

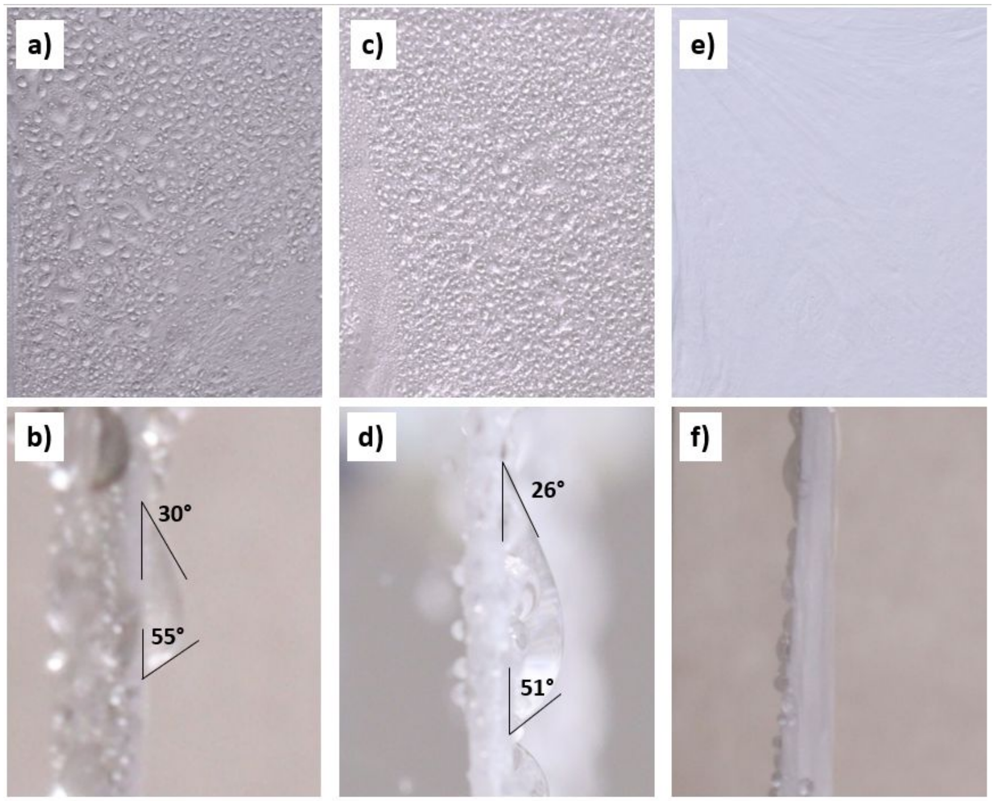

Figure S5. (a) PVDF during 60 min fog harvesting (b) PVDF side profile of droplet contact angle hysteresis [$\Delta\theta$] (c) PVDF-TiO_2-CS-Espray during 60 min fog harvesting (d) PVDF-TiO_2-CS-Espray side profile of droplet CAH (e) PVDF-TiO_2-CS-Espray UV during 60 min fog harvesting (f) PVDF-TiO_2-CS-Espray UV side profile.

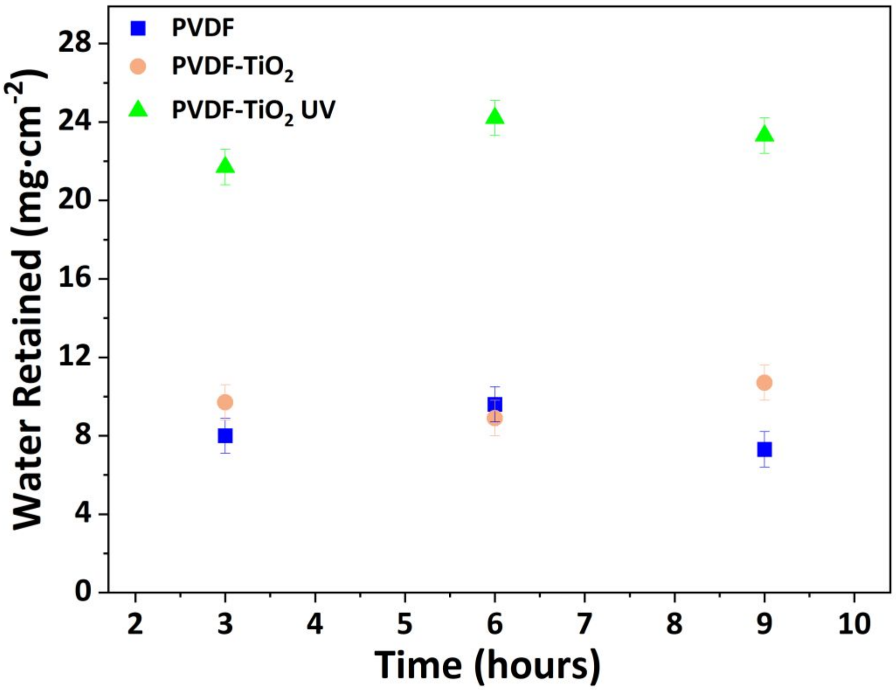

Figure S6. Changes of water retention capacity of fog water with time, up to 10 h.

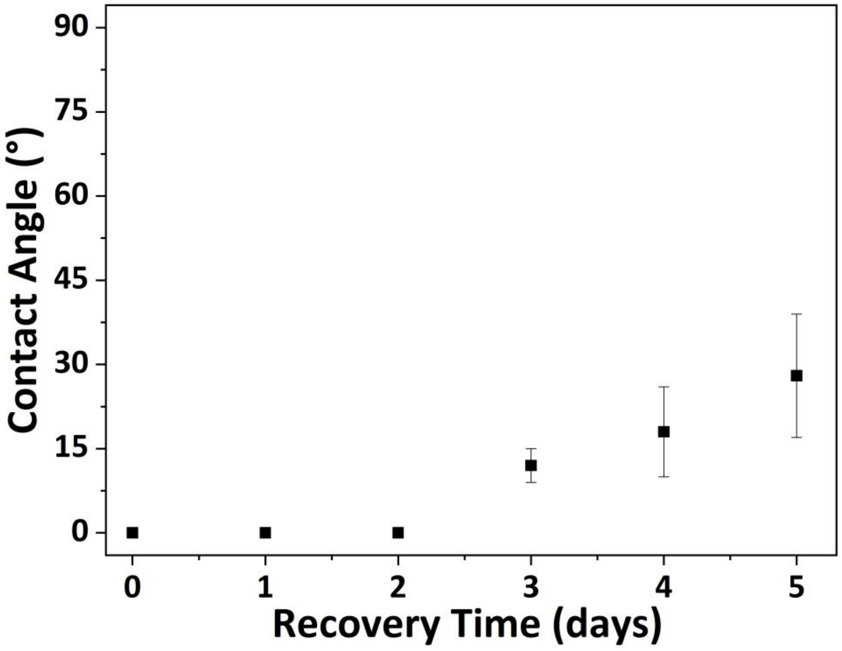

Figure S7. Recovery time of PVDF-TiO_2-Blend-CS-Spray mesh.

Table S1. Average advancing and receding water contact angles and contact angle hysteresis of the 3 electrospun mats used in fog collection experiment.

Fog tested electrospun mats	**Average Advancing contact angle (°)** θ_A	**Average Receding contact angle (°)** θ_R	**Average contact angle hysteresis (°)** $\Delta\theta$
PVDF	58	33	25
PVDF-TiO_2-CS-Espray	52	25	27
PVDF-TiO_2-CS-Espray UV	-	-	-
